# DNA Data Storage

**DOI:** 10.3390/biotech12020044

**Published:** 2023-06-01

**Authors:** Tomasz Buko, Nella Tuczko, Takao Ishikawa

**Affiliations:** Department of Molecular Biology, Institute of Biochemistry, Faculty of Biology, University of Warsaw, Miecznikowa 1, PL-02-096 Warsaw, Poland

**Keywords:** bit, byte, long term data storage, next-generation information storage, oligonucleotide, sequencing

## Abstract

The demand for data storage is growing at an unprecedented rate, and current methods are not sufficient to accommodate such rapid growth due to their cost, space requirements, and energy consumption. Therefore, there is a need for a new, long-lasting data storage medium with high capacity, high data density, and high durability against extreme conditions. DNA is one of the most promising next-generation data carriers, with a storage density of 10¹⁹ bits of data per cubic centimeter, and its three-dimensional structure makes it about eight orders of magnitude denser than other storage media. DNA amplification during PCR or replication during cell proliferation enables the quick and inexpensive copying of vast amounts of data. In addition, DNA can possibly endure millions of years if stored in optimal conditions and dehydrated, making it useful for data storage. Numerous space experiments on microorganisms have also proven their extraordinary durability in extreme conditions, which suggests that DNA could be a durable storage medium for data. Despite some remaining challenges, such as the need to refine methods for the fast and error-free synthesis of oligonucleotides, DNA is a promising candidate for future data storage.

## 1. Introduction

The demand for data storage is increasing by approximately 50% every year. In 2012, the entire world’s total information storage was 2.7 ZB [1], in 2018 it reached 33 ZB, only to rise two-fold in 2020. It is estimated that newly created data will take up about 175 ZB by 2025 [2]. This equals a 65-fold increase only in the period between 2012 and 2025.

The tremendous Global Datasphere expansion is a strong motivator for new developments in data storage. Current data storage methods, such as magnetic (e.g., hard disk), optical (e.g., Blu-ray disc), and solid-state (e.g., flash drive), are insufficient to accommodate such rapid growth [3]. The main problems with those methods are their cost, space, and energy consumption during the recording, storing, and reading of data. Moreover, their durability reaches a maximum of 50 years in perfectly optimal conditions [4]. Humidity, extreme temperatures (both high or low), magnetic fields, or mechanical failures are the main reasons why those methods are not reliable for long-term data storage.

Therefore, there is a great demand for a new, longevous data storage medium with a high capacity, high data density, and high durability against extreme conditions [1]. There are a few prototypes of next-generation data carriers that may be able to cope with the above-mentioned challenges. Among them, DNA seems to be one of the most promising. The most distinguishing features of DNA from other storage media are its density and durability against the extreme conditions.

*Escherichia coli* has a storage density of 10^19^ bits of data per cubic centimeter [5]. This means that 1.7 × 10^19^ bits can be stored in just 1 g of DNA. Due to its three-dimensional structure, DNA is about eight orders of magnitude denser than other storage media. Moreover, DNA replication during PCR or the cell’s proliferation enables the quick and inexpensive copying of vast amounts of data [3].

For years, a DNA specimen collected from a 700,000-year-old horse was considered to be the oldest extracted DNA. However, in 2021, this record was pushed to 1 million years. DNA extracted from mammoth teeth was successfully extracted and sequenced [6]. Additionally, scientists managed to sequence 300,000-year-old mitochondrial DNA from humans and bears [4]. These examples perfectly illustrate the longevity of DNA and proves its usefulness for archeological purposes or data storekeeping. If stored in optimal conditions and dehydrated, DNA can possibly endure for millions of years [1].

Numerous space experiments on microorganisms have proven their extraordinary durability in extreme conditions. Due to solar UV radiance, the space vacuum, and extreme temperature conditions, space is considered one of the most hostile environments [7].

UV radiance being the most deleterious parameter in space increases microorganisms’ lethality by four orders of magnitude in relation to Earth’s conditions [8]. UVB and UVC altogether cover the 200–315 nm light spectrum; these are the most hazardous to microorganisms and are responsible for their high lethality in space. This is caused by high irradiance absorption by DNA and proteins in such spectral ranges. In vegetative cells, this UV irradiance leads to DNA mutations, such as cyclobutane pyrimidine dimers and pyrimidine–pyrimidone photo products [9]. Meanwhile, in bacterial spores, thymine dimer photoproducts, so-called spore photoproducts (SP), are formed due to UV radiation. Despite this fact, all these dimers can be repaired by the direct reversal mechanism. Spores possess an additional SP-specific repair pathway that makes spores significantly more resistant to UV radiance than vegetative cells [10].

Regardless of such hostile conditions, it has been proven that spores of *Bacillus subtilis* shielded against UV solar radiation are able to survive in outer space for nearly 6 years. Although only 1–2% of the population recovered, the outcome was significantly increased (even to 90% of population recovered) if 5% glucose was added to the spore multilayer. It was suggested that glucose binds additional water molecules, preventing the cell from becoming completely desiccated. It also replaces water molecules, thereby stabilizing the macromolecular structure [8]. Furthermore, some microorganisms can even cope with a full space environment. For example, the lichens *Rhizocarpon geographicum* and *Xanthoria elegans* survived a 2-week exposure to outer space. After that time, the lichens completely restored their photosynthetic activity and no ultrastructural changes were revealed in most of the fungal and algal cells of lichens [11]. It is supposed that their thick cortex with UV-screening pigments (rhizocarpic and parietin phenolic acids) are responsible for their survival [12].

## 2. Coding Files in DNA

**Figure 1:**
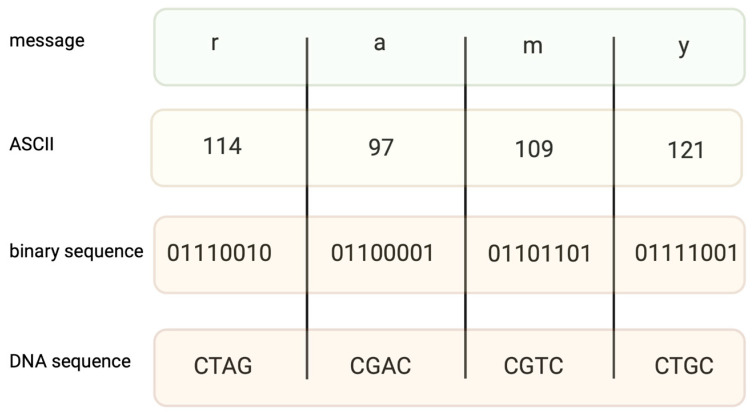
An example of coding the message “ramy” into an ASCII code. Converting binary data into nucleotide sequences is made by computer algorithms.

Encoding information in DNA is based on binary code. A specific nucleotide corresponds to a code, for example, 00 → A, 01 → C, 10 → G, and 11 → T. While binary data are “translated” into a DNA sequence, it is important to avoid long homopolymers (more than three same nucleotides in a row) and unreasonable GC content, as both might generate mistakes during the synthesis and sequencing of DNA strings. In fact, encoding a file requires converting text into a code such as ASCII (Figure 1) or Base64, and then converting the coded file into a binary system. The encoding field uses different coding algorithms, such as Huffman, to condense messages and balance code, preventing homopolymer sequences. Two examples of coding systems, their modifications, and other algorithms of a similar kind generate proper DNA strings [13,14], which are capable of long data storage.

Church et al. (2012), for the first time, encoded a draft of a book, eleven JPG images and one JavaScript program in DNA [15]. For this purpose, they used a simple encoding method involving the translation of zeros into A or C and ones into T or G. As a result, the authors received 54,898 oligonucleotides, each containing three parts: 96 bases of data, 22-bases-long sequences at both ends, allowing those oligonucleotides to be parallelly amplified by PCR, and the 19-bases-long index sequence, pointing out the segment position in the original file [15]. Encoding one bit per base allowed the authors to avoid sequences that were potentially hard to write or read. Splitting information into blocks of data allowed the authors to circumvent the problems associated with the synthesis of long DNA strings. This pioneering work demonstrated the real possibility of using DNA as a data storage material, and also showed the enormous capacity of this method. An important element of the works of that time was to show the limitations of the method used. Through this work, it was noted that the information encoded in DNA is prone to sequencing errors, mainly in homopolymer regions.

**Figure 2:**
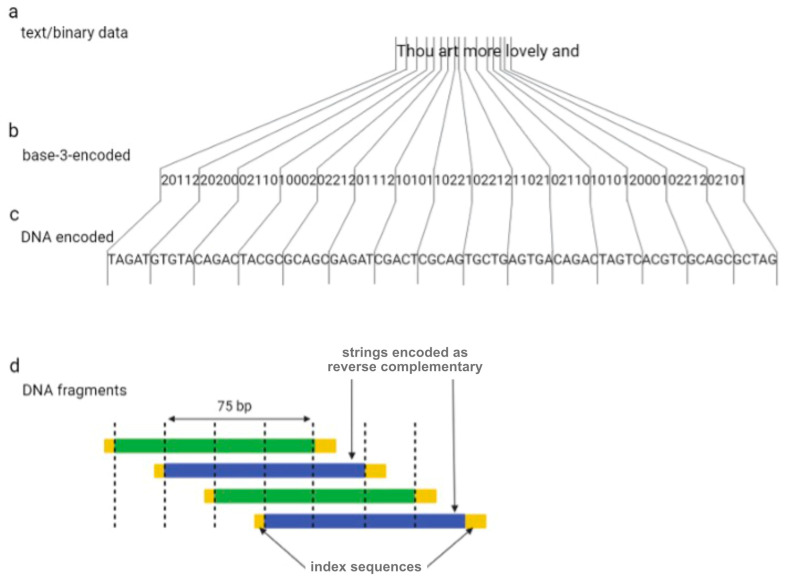
The coding scheme implemented by Goldman et al. Digital information (**a**) is converted to base-3 (**b**) using a Huffman code and is subsequently is converted to DNA strings (**c**). Dividing DNA strings as shown generated four-fold redundancy (**d**).

One year later, Goldman et al. (2013) tried to overcome the sequencing errors occurring by encoding data with redundancy [16]. The authors encoded all 154 of Shakespeare’s sonnets, a scientific article, a medium-resolution color photograph of the European Bioinformatics Institute, and a 26 s long excerpt from Martin Luther King’s 1963 “I have a dream” speech using the Huffman algorithm to covert numeric data into a nucleotide sequence [16]. In summary, bytes of binary sequences were converted into base-3 digits (or ternary) from 0 to 2, which were then associated with three nucleotides, A, T, and C (or G if C has been used for the encoding of the previous ternary digit). DNA strings were divided into 100-nucleotide-long oligos with an overlap of 75 residues between adjacent fragments, creating four-fold redundancy (Figure 2). Alternate fragments were converted to their reverse complement, which reduces the probability of systematic failure, such as issues with DNA sequencing. Indexing sequences comprising 17 nucleotides were also encoded at the beginning and end of each fragment.

**Figure 3:**
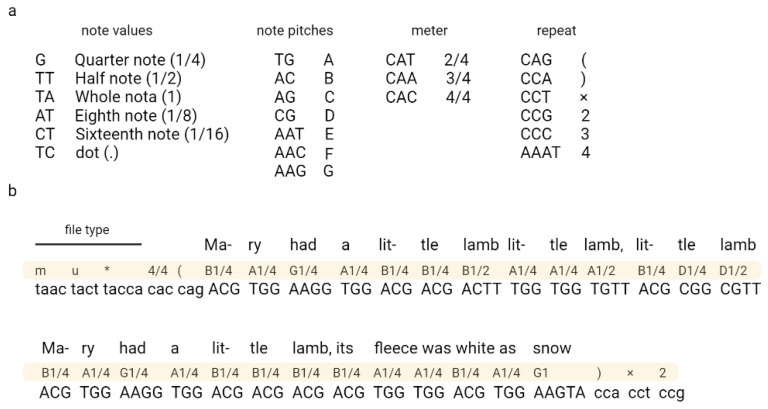
An example of coding music in DNA. Fragment of “Mary Had a Little Lamb” encoded using Huffman code. A nucleotide sequence corresponding to the music code is shown in (**a**) and the encryption part in (**b**). Adapted from Ailenberg and Rotstein [17].

Ailenberg and Rotstein (2009) encoded text, music, and images in DNA by using modified Huffman coding (Figure 3) [17]. In their work, they constructed a plasmids library each containing 10,000 bp of information and an index plasmid that contains basic information, such as the title, author, plasmid number, and primer assignments used to read coded information [17]. The authors also constructed a separate encoding table for each type of file, which allowed the authors to encode each character from the keyboard. The authors also indicated the possibility of extending their code according to the described rules.

**Figure 4:**
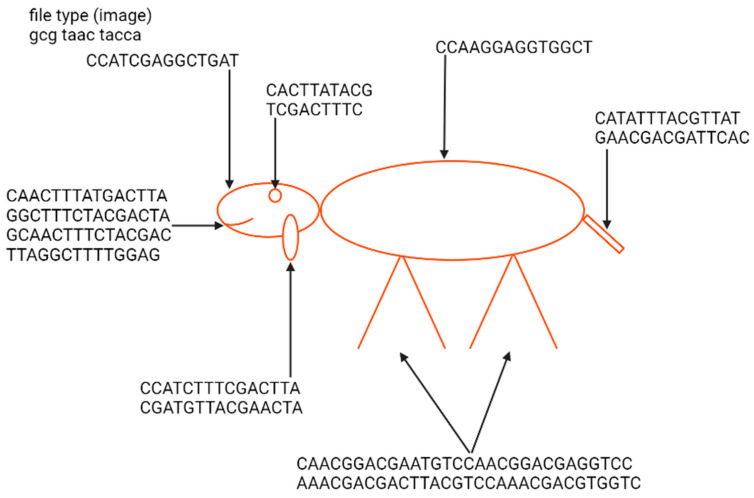
Indication of elements of the nucleotide sequence in which a Little Lamb was encoded and an example image presenting a lamb from the “Mary Had a Little Lamb” rhyme encoded by Ailenberg and Rotstein [17]. The sequence of a file type defines it as an image. The geometric shape of the lamb enables the use of only 238 bp of DNA for encoding. Encoding has been performed using a template of signs indicating the type of shape and its spatial coordinates.

**Figure 5:**
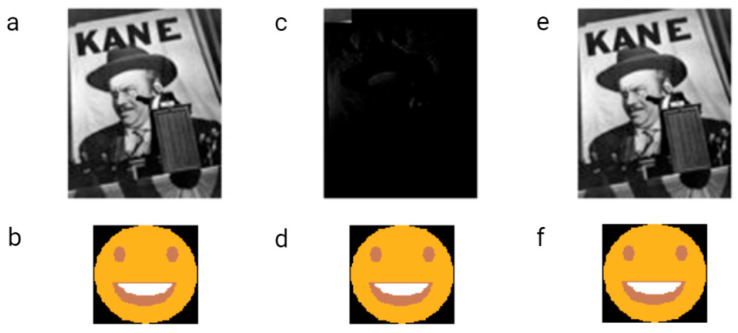
Smiling emoji and original Citizen Kane poster photograph encoded and decoded by Yazdi et al. [18]. The raw images were encoded and synthesized in the form of DNA strings (**a**,**b**). Images received after decoding without homopolymer check codes during processing (**c**,**d**). Images received after sequencing DNA strings when homopolymer error correction was made in order to reduce the number of errors that occurred during each encoding and decoding step (**e**,**f**). Two errors in the Citizen Kane file were sufficient to make the recovery of the image impossible. One error in the emoji did not influence the image quality.

The first example of the graphical file recoded in DNA was a simplified lamb drawing (Figure 4). Although this image consists of simple geometric figures, the simplicity and geometry of the image are not general requirements. Yazdi et al. (2017) managed to encode The Citizen Kane poster photograph and Smiley Face emoji (Figure 5) [18]. For this purpose, they used Base64 encoding to convert files into binary format. The DNA string length used by the authors was 1000 bp, containing 984 bp of information and 16 bp of address sequence. The purpose of the addressing method was to enable random access to codewords via highly selective PCR reactions. This approach allows the specific amplification of a pool of oligos without amplifying and reading all sequences from a given pool. This work also presented a new deletion-correcting method called homopolymer check codes. This method of correction divides DNA sequences into strings of homopolymers, e.g., {AATCCCCGA} into strings {AA, T, CCC, G, A}, which gives a homopolymer sequence of length {2,1,3,1,1}. The homopolymer length sequence contains special redundancy that protects against asymmetric substitution errors. Hypothetically, when two deletions occur in the sequence resulting in {ATCCGA}, the length of the homopolymer fragments is {1,1,2,1,1}. Recovering the original sequence is possible by correcting two bounded magnitude errors. Combining this with GC content balancing, the subsequent alignment of DNA oligonucleotides, and post-sequencing sequence sorting based on the correctness of the index sequence resulted in a new coding method.

Coding motion picture as motion GIFs and movies has also been achieved in the DNA data storage field. In 2017, Shipman et al. encoded five frames of a galloping mare from Eadweard Muybridge’s “The Human and Animal Locomotion Photographs” [19]. In their experiment, CRISPR-Cas was used to integrate an encoded short movie into the genomes of a population of living bacteria. The usage of this method does not change the overall encoding protocol. Strings of DNA are integrated into the CRISPR array thanks to appropriate integrases. Spacer sequences in the CRISPR array were used to encode barcodes defining which set of pixels was encoded in a specific part. The use of the CRISPR method for GIF encoding was of great importance because it allows the encoding of subsequent sequences without the need to additionally index them. This is because newly added sequences are almost always integrated in such a way that they push the previously integrated sequences away from the leader region. Therefore, the order of the sequence was conditioned by successive transformations in which DNA with encoded movie frames was introduced to bacterial cells.

A number of other works referring to information encoding in DNA are summarized in Table 1 below.

## 3. Synthesis of DNA Strings

**Figure 6:**
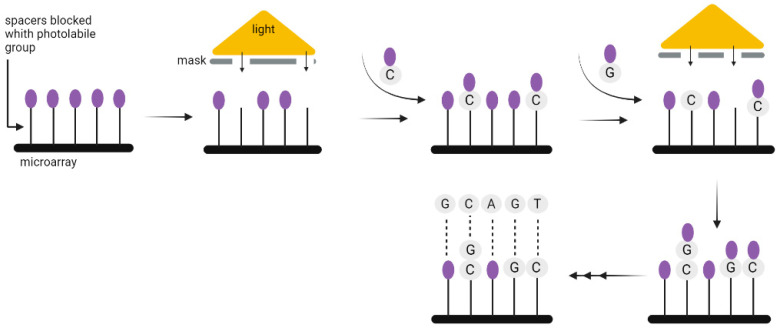
A solid-phase method for the synthesis of oligonucleotides using photolabile compounds. A spacer containing the photolabile group is covalently joined to the surface. Once spots on the surface are exposed to UV light through slits in the physical mask, the photolabile protecting group is removed and the synthesis of oligonucleotide begins. The subsequent appropriate phosphoramidite with the photolabile group is then applied to the entire surface of the plate. It can form covalent bonds only in the absence of the preceding photolabile group. In the subsequent steps, additional spots are exposed to radiation, and another phosphoramidite is applied where necessary. Until the final oligonucleotide is completely synthesized, the chain-extending processes are repeated [29].

Chemical DNA synthesis has made tremendous progress since the 1970s, when fragments of about 20 nucleotides could be synthesized, to the present, when fragments of up to 500 nucleotides can be easily made. The technology commonly used for the synthesis of DNA strands enables only short 200–300 nucleotides sequences to be synthesized, which is a limitation when coding a large amount of data. Nevertheless, the technology used for DNA synthesis on microarrays seems to be more suitable for this purpose. It allows the synthesis of parallel oligonucleotides containing different sequences (Figure 6). By using it, the time and cost needed for the synthesis of large-scale DNA libraries might be greatly reduced [29]. Microarrays have enabled the high-fidelity synthesis of oligo pools of about 300 nucleotides in length [30]. Regardless of the synthesis method, long DNA fragments must be assembled from oligos. It is also necessary to add indexes to each fragment, or sequence overlapping in successive DNA fragments [3], unless—as discussed above—the CRISPR method is used to record information in the bacterial genome. In 2017, Heckel et al. considered the storage capacity using both assembly methods and have shown that an index-based coding system is optimal for data storage purposes [31].

## 4. New Storage Medium, Old Problems, and Solutions

A serious problem with the usage of DNA for data storage purposes is that long-term storage, synthesis, and sequencing might introduce some errors (such as deletion, insertion, or substitution). It should be stressed that errors are not the only issue when DNA is used as the data storage medium, but this is a problem of all information storage technologies. This is why there is a solution to it in the form of error-correcting codes (ECCs), in which a minimal amount of special data is added for error-correction purposes. In classical data-storage devices, the use of ECCs adds redundancy and allows the correction of essentially all errors that occur during use. ECCs such as fountain code, rapid tornado code, HEDGES (Hash Encoded, Decoded by Greedy Exhaustive Search), or the Reed–Solomon code [32] are used in DNA data storage. In general, ECCs introduce sequence redundancy, which enables the subsequent recovery of complete data even in the case that some oligonucleotides used for data storage are physically damaged. The implementation of ECCs slightly diminishes the storage capacity (because ECCs are often based on adding external fragments to the sequences encoding data), but its advantages—namely the possibility of error correction—outweigh this limitation. ECCs enable insertions and deletions to be corrected, as well as the loss of some parts of the DNA strings. An alternative to ECCs was the previously used high-depth sequencing, which, for obvious reasons, only corrected sequencing errors.

**Figure 7:**
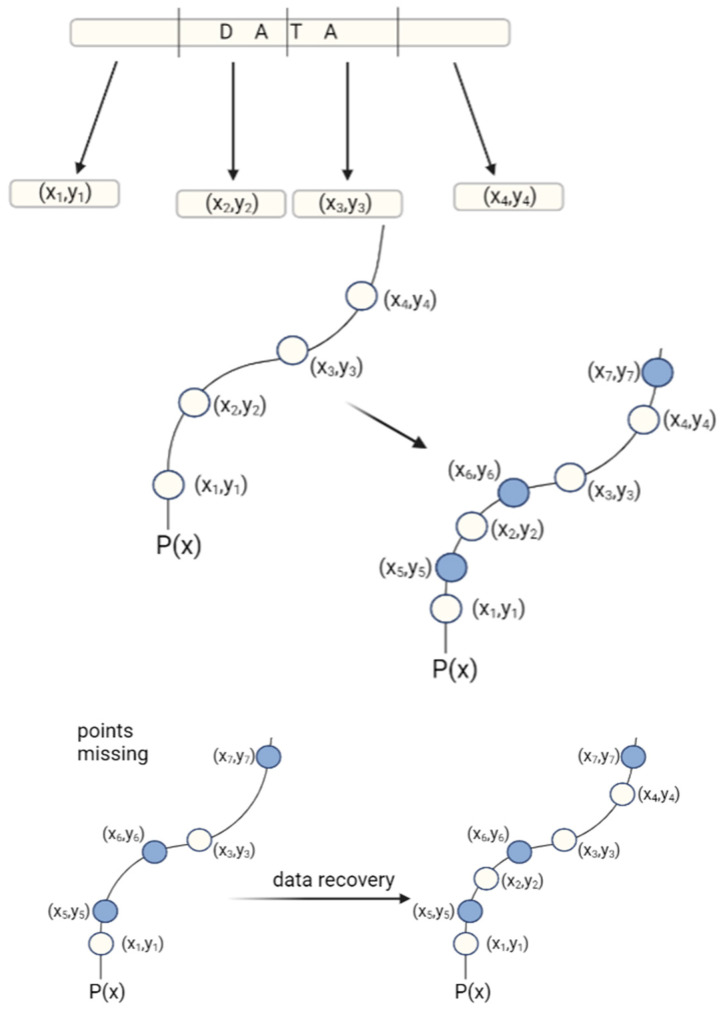
Principle of Reed–Solomon correction: first, the data is divided into parts, and each part is assigned x and y values that determine its location. Based on the coordinates, the points are matched to the polynomial function P(x), which is used to determine the parity symbols. Parity symbols are extra data points that match the original DNA sequence and are stored with the original data. When some of the original data are lost, the remaining data points and parity symbols can be used to recreate the original polynomial function and receive original data.

One of the most frequently mentioned ECCs in the literature is a Reed–Solomon code (Figure 7). In general, the Reed–Solomon code is based on the transformation of the original data set to a symbol set. The symbols are then converted to coefficients in a system of linear equations and their solutions enable the original data set to be accessed. Meiser et al. (2020) have used a Reed–Solomon code for storing a full album of music in DNA [33].

Recently, Xie et al. (2023) conducted an analysis showing the value of the sequencing depth for retrieving the right string of data [34]. Sufficiently deep sequencing allows the use of MSA (multiple sequence alignment) methods to establish a consensus sequence and correct errors that may appear on the DNA strands. The MAFFT algorithm was chosen for the analysis, which has been shown to be able to correct more than 95% of errors at a sequencing depth reaching 100× when the error rate is lower than 15%. The authors showed that adequately deep sequencing combined with MSA is able to correct errors when their frequency is less than 20%. Above this value, error correction based on MSA is possible with the simultaneous use of ECC. This method enables the cost and time reduction needed for the DNA data storage procedure. 

**Figure 8:**
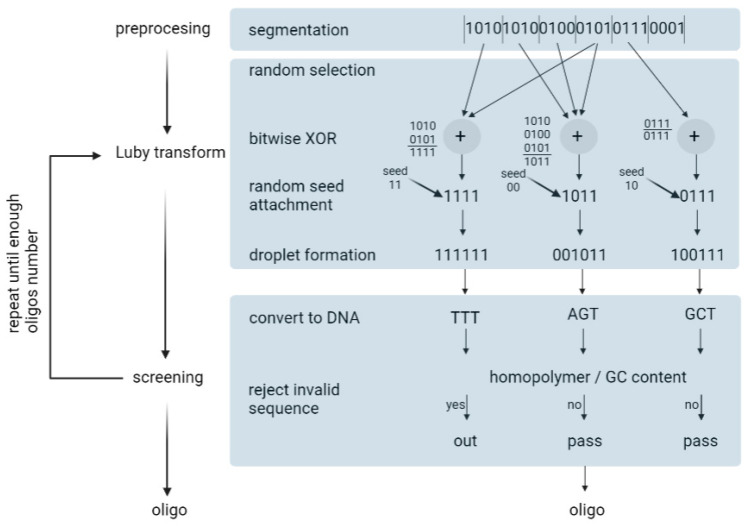
Depiction of DNA fountain strategy.

Erlich and Zielinski (2017) used the fountain algorithm to encode 2.14 × 10^6^ bytes of data [35]. The fountain encoding algorithm works in three steps: preprocessing, the Luby transform, and screening (Figure 8). Overall, it aims to convert the input file into a collection of DNA strings that pass synthesis and reading constraints.

*Preprocessing*—In this step, the input file is compressed using a lossless algorithm. Then, the algorithm partitions the file into non-overlapping K segments, in which each segment is L bits long. L is defined by the user.
*Luby transformation*—This step consists of many substeps. Briefly, a pseudo-random number generator determines the number of segments that will be packed into a single packet. Encoded segments become packets known as droplets. For this, the algorithm uses a robust solution probability distribution, which assumes that most of the droplets will be created with a small number of input segments. On the segments of one droplet, the algorithm performs a bitwise exclusive or XOR operation. For example, consider that the algorithm randomly selected three input fragments: 0100, 1100, 1001. In this case, the droplet is 0100 ⊕1100 ⊕1001 = 0001. In the end, the algorithm adds an index that specifies the binary representation of the seed, which, in turn, corresponds to the state of the random number generator of the transform during the generation of the droplet. Finally, it enables the decoder algorithm to infer the identities of the segments in the droplet.
*Screening*—In the last step, the algorithm excludes those strings that do not pass the biochemical constraints. Firstly, binary data are translated into a nucleotide sequence: {00, 01, 10, 11} to {A, C, G, T}. Then, DNA strings are screened for GC content and homopolymers. The sequences that do not pass the screen are removed and the formation and screening of the oligonucleotides are repeated until the desired conditions are obtained. In practice, the authors recommend synthesizing 5–10% more oligonucleotides than the input segments.

The idea for the decoding algorithm is to start with single-segment droplets and propagate that information through the other droplets until all the segments are recovered.

## 5. DNA Preservation

Although the theoretical density of DNA data storage reaches petabytes per gram, usually this value is unreachable. Due to the necessity of adding protective substances to the DNA, the loading efficiency (DNA weight/total weight) ranks below 100%. Moreover, the presence of indexes, such as Reed–Solomon codes, in long strands of DNA cause the loss of data storage density. It was estimated that the index ratio of 200 bp DNA reaches 6.5%. Furthermore, DNA without protection is liable to degradation due to physical and chemical factors, such as temperature, water, UV irradiation, oxidation, or extreme pH values [36]. Therefore, current research focuses on increasing the DNA data storage density and the time of its preservation by protecting DNA from the influence of high humidity and the presence of oxygen [37].

The methods used for DNA preservation can be divided into two essential categories: in vitro preservation, where DNA is usually stored in a single physical DNA pool, or in vivo preservation, which uses living cells as DNA carrier systems [32].

### 5.1. In Vitro Preservation

The most common way to store data within DNA in vitro is solution storage. At first, DNA was preserved in ethanol, however, over time the ammonium-based ionic liquids gained popularity. Due to hydrogen bonding between ionic liquid and DNA, those solutions improve DNA stability. However, the solution storage allows DNA to be stored for only a year, which is insufficient to fulfill the aims of DNA data preservation (>1000 years).

On the contrary, solid-state DNA appears to be more stable due to its reduced molecular mobility and lack of water, which causes hydrolytic damage [35]. The successful amplification of DNA from ancient specimens, such as the Pleistocene cave bear, additionally indicates the effectiveness of the method [37]. Based on this discovery, Grass and co-workers proposed DNA silica fossilization technology, through which they obtained stable DNA after 35 days in 65 °C (equivalent to two years at room temperature) [38]. Furthermore, Newman et al. (2019) developed a method for the preservation of dehydrated DNA spots on glass cartridges, which can subsequently be recovered by a water droplet. Multiple DNA spots on one cartridge additionally increase the storage density of 50 TB of data per glass cartridge [39]. Choi et al. (2020) created a DNA micro-disc, which allows easy access to data-encoded DNA and write-once-read-many memory. Firstly, the encoded DNA’s primer sequences and data description were included in the QR code, which facilitates easy access to the data. Secondly, due to the immobilization of DNA on the micro-disc, after DNA enrichment using PCR, the original and amplified DNA are separated. The sequence of the amplified DNA is subsequently converted into binary data and the immobilized DNA can be read out in the future. Eventually, Choi et al. (2020) reached a density of up to 10^12^ bit/mm^3^ for a single micro-disc and assessed the durability of dehydrated DNA over 100 years at a temperature below 10 °C [40].

DNA can also be easily stored via freeze drying or the addition of additives. In fact, the lower the temperature, the longer the possible preservation. However, lyophilization may cause cytolysis due to the formation of ice cracks [36]. Moreover, the estimated annual cost of maintaining frozen samples around the globe likely surpasses USD 100 million each year [41]. Therefore, due to the high cost currently, scientists are trying to develop an effective method of DNA preservation at room temperature. For instance, the addition of additives such as trehalose or PVA enables the DNA to be preserved at room temperature. Both stabilizers create hydrogen bonds with negatively charged phosphate groups in DNA, which has a protective effect on its stability [36]. However, Ivanowa and Kuzmina (2013) indicate that, generally, the additives are insufficient for long-term DNA storage. Diluted DNA in trehalose solution stored for a month at room temperature granted only 46% PCR success, and 2-year preservation in Tris-buffered PVA granted 50% PCR success, where PCR success was calculated as a percentage of positive wells per plate (96 samples) [42].

In Table 2, we summarize the storage methods used and the PCR success after storage for a specified period at a specified temperature.

In Table 3, we present the durability of DNA in various accelerated aging tests. Such tests are performed to simulate the long-term behavior of DNA molecules in a much shorter time by applying harsh conditions. The results of those experiments are presented as C/C_0_ (%), which is the percentage of the initial amount of DNA present in the sample after the accelerated aging test.

### 5.2. In Vivo Preservation

Recently, in vivo preservation has been intensively developed. Preservation within a living cell allows the DNA to be replicated with a few orders of magnitude, much faster than by PCR, during the cell’s proliferation processes [67].

Bacteria are the most intuitive way to preserve DNA within a living organism. However, during bacterial replication, the spontaneous mutation rate is 2.2 × 10^−10^ mutations per nucleotide per generation, or 1.0 × 10^−3^ mutations per genome per generation [68]. A generation time of about 20–30 min for *E. coli* means that after a few years of cultivation, mutations might represent a significant problem. Furthermore, the size of the introduced plasmid is a serious limitation of in vivo preservation methods. So far, the greatest amount of information in vivo has been encoded by Hao et al. (2020) thanks to the mixed-circle method developed by them. The procedure involves the cloning of data-encoded DNA oligonucleotides into plasmids and transforming *E. coli* cells with recombinant, data-containing plasmids. During data recovery, plasmids are sequenced, and oligonucleotides are assembled into original sequence. Eventually, 2304 kbp synthetic oligonucleotides (encoding 455 KB of digital files) were used to create the mixed culture of bacterial cells [67].

The solution to the problem of the limited size of the introduced plasmid appears to be in vivo preservation on a yeast artificial chromosome. In 2021, Chen et al. created a circular 255 kbp yeast artificial chromosome (a data-carrying chromosome; dChr) encoding a total of 38 KB of digital data (two pictures and a video) [69]. Moreover, the dChr was replicated with high fidelity, no mutation appeared after the 100th generation of replication, while the encoding method used in this setup was tolerant toward a comparatively low accuracy of Nanopore sequencing, enabling the fast retrieval of reliable data [69]. The high fidelity of dChr replication could be achieved due to its chromatin-like structure formed in vivo [70]. As it is known that nucleosomes regulate DNA repair mechanisms [71,72], the utilization of eukaryotic organisms, such as *Saccharomyces cerevisiae*, carrying dChr is one of the promising approaches for DNA data storage. 

Another approach to in vivo storage is the preservation of data in endogenous DNA, such as genomic DNA. This can be achieved using DNA-modifying enzymes such as nucleases, integrases, or recombinases, although recently, the CRISPR-Cas9 system has gained much popularity [73]. At the beginning of 2022, Liu et al. used a dual-plasmid system based on a single crRNA-guided endonuclease (CRISPR-Cas12a) to encode a codebook (56 bytes) and a picture (376 bytes) [74]. The authors used two plasmids, one with data-encoded (target) DNA and the second with templates for the expression of Cas protein and crRNA, which after bacteria transformation, enabled the introduction of target DNA to the *E. coli* genome. Ultimately, the rewriting reliability reached 94% and the information sequenced from the 252nd generation was 100% correct [74].

Studies on antimutator phenotypes have provided valuable insights into the sources and mechanisms of spontaneous mutations. Research on carbon-starved *E. coli* populations has shown that stress responses are required for the mutagenic repair of DNA breaks [75]. In the growing *E. coli* population, mutants of the α subunit of replicative DNA polymerase III have been well characterized as antimutator alleles, suggesting that DNA replication errors are a major source of spontaneous mutagenesis under optimal growth conditions [76]. However, these alleles also reduce specific transition mutations, making it unclear whether replication errors in wild-type cells stem from the intrinsic fidelity of DNA polymerase III or specific subpopulations with unique properties [77].

Despite the understanding of the molecular mechanisms controlling mutagenesis, the process of spontaneous mutation in cells with functional mutation-prevention systems remains unknown. To investigate this, a mutation assay on isogenic *E. coli* cells growing optimally without external stress was performed. It was revealed that spontaneous DNA replication errors occurred more frequently in subpopulations experiencing internal stresses, such as issues with proteostasis, genome maintenance, and reactive oxidative species production. These mutator subpopulations do not significantly impact the average mutation frequency or the overall fitness of the population in a stable environment. However, they play a crucial role in enhancing population adaptability in fluctuating environments by providing a reservoir of increased genetic variability [78].

In turn, such mutator subpopulations may be responsible for introducing spontaneous mutations in the *E. coli* population used for DNA data storage. Further understanding the molecular background of spontaneous mutations may be helpful in minimizing the occurrence of errors in the DNA used as a data storage medium in in vivo preservation methods.

## 6. DNA Sequencing

To convert the DNA sequence back to its digital code, DNA has to be sequenced and decoded to digital data using computer algorithms. Currently, the most commonly used platforms for the sequencing of data-encoding DNA are Next-Generation Sequencing by Illumina sequencing and Third Generation Sequencing by Oxford Nanopore Technology [37].

One of the biggest advantages of Nanopore over Illumina for data output purposes is its single-molecule sequencing of the extended alphabet, or its ability to sequence not only natural nucleotides, but also chemically modified nucleotides. The applicability of such an extended alphabet could significantly improve data storage in DNA by increasing storage density and, possibly, writing speed [79]. However, Nanopore also has some limitations, for instance, lower accuracy compared to Illumina. In fact, a direct comparison of the error rates of Nanopore (∼10% per nucleotide in single read-out) and of Illumina (∼0.5% per nucleotide) shows that Nanopore technology is approximately 20 times less accurate. Therefore, at the moment, for DNA data storage purposes, the most commonly used is Illumina sequencing [37].

## 7. Conclusions

Modern societies generate huge amounts of data and the rate of their growth has multiplied in recent years. The need to store both currently generated data and those generated in the past using classical data storage methods are consuming huge financial outlay and physical space. It also entails high costs for the environment, with the introduction of new methods of data storage thus urgently required.

For a long time, people have paid attention to the high storage density and longevity of DNA. In this article, we have provided a brief overview of how information is encoded and stored in DNA. The continuous development of these methods leads to a reduction in the number of errors appearing in the encoding and decoding processes, extending the durability of DNA as a data carrier, and reducing the cost of its storage.

Despite the continued growth in the field of information storage on DNA, some challenges still remain. There is a need to refine the methods used for the fast and error-free synthesis of oligonucleotides, and in the long run, also of long DNA chains. The method used to read nucleotide sequences also must evolve towards greater credibility.

Despite the current obstacles, the prospects for implementing data storage on DNA are very promising. There are even new ideas related to the use of chemical analogues of DNA, such as TNA, with even higher possible storage densities [26].

## Figures and Tables

**Table 1:** Works regarding the coding of information on DNA. In “redundancy or error correction” column, “n.d.” indicates that there is no information in the original work.

Authors	Data Size	Length of Strings	Encoding Method	Redundancy or Error Correction	Modification	Reference
Bornholt et al.	51 KB	120	Huffman code	DNA string exclusive-or	–	[20]
Blawat et al.	22 MB	230	Own bit mapping	BCH code	–	[21]
Organick et al.	200 MB	~150	Base-4	Reed–Solomon	–	[22]
Choi et al.	854 B	85	Own bit mapping	Reed–Solomon	Degenerate bases	[23]
Lee et al.	96 B	~50	ASCII	codec	Enzymatic DNA synthesis	[24]
Tabatabaei et al.	2 KB; 392 KB	450	Own bit mapping	Not needed	Enzymatic nicking (*Pf* Ago)	[25]
Yang et al.	23 KB	83	A, C = 0; G, T = 1	n.d.	TNA	[26]
Ren et al.	682 B; 39 KB; 28 MB	~100	RABR; RALR	Reed–Solomon	Artificial nucleotides	[27]
Mayer et al.	24,5–33,6 KB	~40	ASCII; Elias gamma	n.d.	Epigenetic encoding	[28]

**Table 2:** Storage methods of DNA and PCR success after the storage.

Storage Method	Time	Temperature	PCR Success	Reference
Chemical encapsulation				
Silica nanoparticles	9 months	RT	x	[43]
DNA-layered titanate nanohybrid	1 month	x	x	[44]
Solution Preservation				
”DNA stable”	4 years	RT	98%	[42]
DMSO salt solution	4 months	RT	42%	[45]
DMSO salt solution	2 years	RT	x	[46]
70% ethanol	4 months	RT	27%	[45]
70% ethanol	2 years	RT	x	[46]
90% ethanol	6 months	RT	96%	[47]
Formalin-fixed	30 years	RT	30%	[48]
Formalin-fixed	2–6 years	RT	x	[49]
Paraffin-embedded tissues	2–6 years	RT	x	[49]
DETs buffer	6 months	RT	92%	[47]
TE buffer	1 night	−20 °C	100%	[50]
TE buffer	3 years	−20 °C	x	[51]
Dehydratation				
Ancient bone	521 years	13 °C	x	[52]
Filter Paper	4 years	RT	82.5%	[53]
Dried DNA	4 months	RT	35%	[45]
FTA cards	up to 128 days	RT	95%	[54]
Silica Gel	6 months	RT	50%	[47]
Oven-dried	6 months	RT	72%	[47]
Oven-dried	6 months	−20 °C	86%	[47]
Freeze drying				
DNA	4 years	4 °C	49%	[42]

RT is abbreviation for “room temperature”. X indicates that the information was not specified in the reference.

**Table 3:** The durability of DNA in accelerated aging tests.

Storage Method	Time	Temperature	Relative Humidity	Half-Life	Temperature	C/C_0_	Reference
	Experimental Conditions			Parameters in Non-Experimental Conditions			
Chemical encapsulation							
Silica nanoparticles	2 weeks	70 °C	50%	20–90 years	20 °C	90%	[43]
Silica nanoparticles	10 days	60 °C	50%	5 months	RT	65%	[55]
Calcium phosphate crystals	6 days	70 °C	50%	1 year	10 °C	0.1%	[56]
”DNAshell”	2 days	100 °C	50%	1 million years	25 °C	x	[57]
”DNAshell”	30 h	76 °C	50%	100 years	25 °C	x	[58]
”DNAshell” + trehalose	1 month	76 °C	50%	2000 years	25 °C	x	[58]
In silica	1 week	70 °C	50%	200 years	10 °C	10%	[4]
Solution Preservation							
”DNA stable”	1 week	65 °C	50%	4 years	25 °C	10%	[4]
”GenTra”	1 week	65 °C	50%	2 years	25 °C	50%	[59]
TE buffer	20 days	65 °C	50%	20 years	−20 °C	x	[51]
Dehydratation							
DNA	6 weeks	50 °C	50%	x	x	10%	[60]
DNA silica fossilization	35 days	65 °C	50%	2 years	RT	15%	[61]
Dehydration with earth alkaline salts	6 days	70 °C	50%	750 years	10 °C	10%	[62]
DNA micro-disc	2 weeks	70 °C	50%	>700 years	0 °C	x	[40]
DNA with trehalose	10 days	70 °C	75%	17 years	10 °C	x	[63]
Filter card	1 week	70 °C	50%	3.7 years	25 °C	1%	[4]
Freeze drying							
Polymer-plasmid complexes	10 months	40 °C	50%	3 years	RT	x	[64]
Trehalose	2 months	60 °C	50%	2 years	RT	x	[65]
Cryosilicified samples	4 weeks	70 °C	60%	1200 years	20 °C	31%	[66]
Additives							
Trehalose	2 years	56 °C	50%	20 years	RT	50%	[42]
Trehalose	1 week	65 °C	50%	160 years	10 °C	20%	[59]
PVA	2 years	56 °C	50%	20 years	RT	15%	[42]
”Sugar mix”	1 week	65 °C	50%	1 year	20 °C	30%	[59]

RT is abbreviation for “room temperature”. x indicates that the information was not specified in the reference.

## Data Availability

Not applicable.
